# Fundamental prognostic difference of *ATM* gene mutation and deletion in newly diagnosed mantle cell lymphoma

**DOI:** 10.1186/s10020-025-01376-2

**Published:** 2025-09-29

**Authors:** Ales Obr, Diana Malarikova, Eva Kriegova, Helena Urbankova, Zuzana Zemanova, Jirina Manakova, Anna Petrackova, Michaela Vatolikova, Adela Berkova, Kristina Forsterova, Tomas Furst, Andrea Hruskova, Patrik Flodr, Veronika Hanackova, Vit Prochazka, Tomas Papajik, Marek Trneny, Pavel Klener

**Affiliations:** 1 Department of Hemato-Oncology, Faculty of Medicine and Dentistry, Palacky University and University Hospital, Olomouc, Czech Republic; 2 First Department of Internal Medicine, Department of Hematology, First Faculty of Medicine, University General Hospital, Charles University, Prague, Czech Republic; 3 Department of Immunology, Faculty of Medicine and Dentistry, Palacky University and University Hospital, Olomouc, Czech Republic; 4 Centre of Oncocytogenomics, Institute of Medical Biochemistry and Laboratory Diagnostics, First Faculty of Medicine, General University Hospital, Charles University, Prague, Czech Republic; 5 Department of Mathematical Analysis and Applications of Mathematics, Faculty of Science, Palacky University, Olomouc, Czech Republic; 6 Department of Clinical and Molecular Pathology, Faculty of Medicine and Dentistry, Palacky University and University Hospital, Olomouc, Czech Republic; 7 Institute of Pathological Physiology, First Faculty of Medicine, Charles University, Prague, Czech Republic

**Keywords:** Mantle cell lymphoma, ATM, Deletion, Mutation, Survival

## Abstract

**Background:**

Previous studies have suggested that, after acquisition of t(11;14), mantle cell lymphoma (MCL) pathogenesis may proceed via several different genetic second hits, which may shape different mutational profiles of clinically manifest lymphoma. The most prevalent second hit in MCL includes *ATM* aberrations, accounting for about half of patients with newly diagnosed MCL. As *ATM* and *TP53* mutations tend to be exclusive in MCL, we retrospectively analyzed the prognostic role of *ATM* deletions and/or mutations in patients with newly diagnosed MCL, both in the entire cohort and in a subcohort of patients with wild-type *TP53*.

**Methods:**

To investigate deletions and mutations of *ATM* and *TP53* in newly diagnosed MCL, we used fluorescence in situ hybridization and next-generation sequencing. To assess relationships between variables, non-parametric (Spearman) and chi-square tests were used. The Kruskal–Wallis test was used to analyze differences in continuous variables between two groups of patients. For survival analyses, the standard Kaplan–Meier estimator and log-rank test were employed. Univariate and multivariate Cox proportional hazard models were used to examine the prognostic value of various factors on patient survival.

**Results:**

We analyzed 187 patients with MCL (a median follow-up of 3.6 years). Eighty-one (43%) and 75 (40%) patients had *ATM* and *TP53* aberrations, respectively. Of note, three (9%) patients with mutated *ATM* harbored a germline mutation. Patients with *TP53* aberration had shorter survival rates. Although *ATM* deletion did not correlate with progression-free survival (PFS) in the entire cohort, it was associated with shorter PFS (hazard ratio 2.25, *p* = 0.01) in patients with wild-type *TP53*. A higher frequency of *ATM* deletion correlated with shorter PFS. Patients with *ATM* mutation (and wild-type *TP53*) had a trend toward better PFS (albeit not statistically significant). Moreover, patients with a higher variant allele frequency of *ATM* mutation tended to have longer PFS.

**Conclusions:**

*ATM* deletion is an important predictor of prognosis in MCL patients and should be routinely examined, especially in those with wild-type *TP53*. In contrast, an isolated *ATM* mutation may predict a better prognosis in the context of standard immunochemotherapy.

**Supplementary Information:**

The online version contains supplementary material available at 10.1186/s10020-025-01376-2.

## Background

Mantle cell lymphoma (MCL) is a relatively rare subtype of B-cell non-Hodgkin lymphoma, with an incidence of one to two cases per 100,000 people per year. Most patients are male, with a median age at diagnosis of 65 years. Eighty to 90% of patients with classic MCL have an aggressive clinical course, with a historically short median survival of up to seven years (Dreyling et al. 2017; Herrmann et al. 2009; Salek et al. 2014; Obr et al. 2019). In contrast, the minority of patients with non-nodal MCL tend to have an indolent presentation and dramatically better prognosis (Alaggio 2022).

MCL is defined by the hallmark chromosomal translocation t(11;14)(q13;q32), which juxtaposes the *CCND1* (*BCL1*) gene to the immunoglobulin heavy chain locus, resulting in the overexpression of cyclin D1. Previous studies have suggested that, after acquisition of t(11;14), MCL pathogenesis may proceed via several different genetic second hits, which may shape different mutational profiles of clinically manifest lymphoma. The most prevalent second hit in MCL includes ataxia-telangiectasia mutated (*ATM*) aberrations, accounting for approximately 50% of patients with newly diagnosed MCL (Malarikova et al. 2020; Camacho et al. 2002; Mareckova et al. 2019; Karolová et al. 2023; Greiner et al. 2006; Ahmed et al. 2016). This serine/threonine protein kinase interacts with the p53 protein and reacts to DNA alterations by activating DNA damage checkpoints, consequently inhibiting the cell cycle, DNA repair, and apoptosis (Lowndes and Murguia 2000; Shiloh 2001; Stakyte et al. 2021). Aberrations in the*ATM* gene, involving deletions, insertions, and base substitutions, lead to a deficiency in the DNA-damage response. The gene was named after ataxia-telangiectasia (AT; Louis-Bar syndrome), a severe hereditary disease caused by a defect in both alleles of the *ATM*gene (Savitsky et al. 1995). Patients with AT have, among other pathological signs and symptoms, an increased risk of developing lymphoma and leukemia. Moreover, the population with*ATM*aberrations is more predisposed to breast, prostate, stomach, and other cancers (Reiman et al. 2011; Choi et al. 2016).

One of the most significant negative predictors of prognosis in MCL is a mutation or deletion of the tumor protein p53 (*TP53*) gene, located on chromosome 17p13.1. The occurrence of *TP53*gene disruption in MCL is approximately 22%, based on a recent meta-analysis of several reports (López et al. 2024).

The *TP53* gene is responsible for the cellular response to various stress stimuli and plays a crucial role in cancer prevention. Both deletion (del17) and mutation of *TP53* (mut*TP53*) are associated with an unsatisfactory treatment response and shorter survival (Malarikova et al. 2020; Sakhdari et al. 2019; Eskelund et al. 2017; Obr 2020; Obr et al. 2018).

Although aberrations of the *ATM* gene are common in MCL, data on its impact on survival are still limited. Most published results thus far have not found a significant association between *ATM*gene disruption and survival (Camacho et al. 2002; Mareckova et al. 2019; Greiner et al. 2006; Salaverria et al. 2007). On the other hand, a few studies suggest a potentially dismal outcome for MCL with*ATM*aberrations (Che et al. 2023; Webb et al. 2023).

The present study analyzed the prognostic significance of *ATM* gene aberrations in a large cohort of MCL patients treated at two university centers in the Czech Republic. We also focused on the prognostic and predictive impact of *ATM* mutational burden and the frequency of *ATM* deletions.

## Materials and methods

### Patient selection

The study cohort consisted of 187 consecutive adult patients newly diagnosed with MCL and treated at University Hospital Olomouc and University General Hospital Prague, Czech Republic. All patients were diagnosed between April 2006 and October 2022, and the median follow-up period was 3.6 years. The study was approved by the respective institutional review boards; all patients provided informed consent and were treated according to the Declaration of Helsinki. All MCL diagnoses were made and reviewed by a reference hematopathologist following the 5th edition of the World Health Organization Classification of Haematolymphoid Tumours: Lymphoid Neoplasms (Alaggio et al. 2022). The patient database was last updated on February 23, 2023. Bone marrow (BM) or peripheral blood (PB) with tumor involvement of ≥ 5% by flow cytometry was available from all analyzed patients. Fluorescence in situ hybridization (FISH) and next-generation sequencing (NGS) were used for the detection of*TP53* and *ATM* gene deletions and mutations, respectively. To prove the germline origin of the *ATM* mutation, buccal swabs were tested.

### Fluorescence in situ hybridization

Cells obtained from complete PB or BM were examined at the time of diagnosis. Samples were cultured for 24 h (BM) or 72 h (PB) in RPMI 1640 medium or BM medium (Gibco, USA; Biological Industries, USA). PB cells were stimulated to proliferate in culture with 12-O-tetradecanoylphorbol-13-acetate (Sigma Aldrich, Germany) or in the presence of DSP30 (TIB Molbiol, Germany) or ECAMPO10 (Euroclone, Italy) with interleukin 2 (Sigma Aldrich, Germany). Cell suspensions were prepared using a standard cytogenetic protocol and stored in Carnoy’s solution in a refrigerator during testing; later, they were frozen at −20 °C. The copy number of the *ATM* and *TP53* genes was determined using FISH with the commercially available probes XL TP53/17cen and XL ATM/11cen (MetaSystems, Germany) according to the manufacturer’s instructions. Hybridization results were evaluated using the Olympus BX60 or Zeiss Imager Z2 fluorescence microscope and scanned with the ISIS software (MetaSystems). Two hundred nuclei of interphase cells and at least 20 metaphases (if present) were evaluated in each hybridization. Gene deletion was expressed as the percentage of cells with one copy of the region of interest relative to the total number of cells evaluated. To avoid false-positive results due to technical limitations of the method, a 5% cut-off level was established.

### Array comparative genome hybridization

Array comparative genomic hybridization (CGH) was performed using SurePrint G3 ISCA CGH + SNP Microarray Kit, 4 × 180 K (Agilent) according to the manufacturer’s protocol. Results were evaluated with Cytogenomics software (Agilent) according to Genome Reference Consortium Human Build 37. Array CGH was used to accurately determine the number and size of deleted 11q regions.

### Next-generation sequencing

Targeted deep NGS assessment of the *ATM*gene coding sequence (exons 2–63 including a 10 bp intronic overlap; NM_000051) was performed as a paired-end on MiSeq (2 × 151 bp, Illumina) with a target read depth of 2,000x. The detection limit was 3% variant allele frequency (VAF). All detected sequence variants were manually screened using the Integrative Genomics Viewer and annotated using clinical databases/tools (COSMIC, ClinVar, Ensembl Variant Effect Predictor), after excluding sequence variants present in 1% of the population (ExAC, gnomAD, 1000 Genomes Project). Only pathogenic or likely pathogenic variants were reported, as assessed by the ACMG criteria (Richards et al. 2015). The*ATM* mutational burden was defined as the VAF of mutations detected, or the highest VAF in the case of multiple mutations present in a single patient.

### Statistical analysis

Overall survival (OS) was defined as the time from diagnosis to the date of the last follow-up (censoring) or the date of death (event) from any cause. Progression-free survival (PFS) was defined as the time from diagnosis to the last follow-up (censoring) or the date of relapse, progression, or death from any cause (event). To assess the association between two continuous variables, the non-parametric (Spearman) correlation coefficient was used. To assess the relationship between two categorical variables (e.g., gender composition of two groups of patients), the chi-square test in a contingency table was used. For testing the difference in continuous variables between two groups of patients, the Kruskal–Wallis test was used.

The Kruskal–Wallis test is a non-parametric method of testing whether samples originate from the same distribution. In survival analysis, the standard Kaplan–Meier estimator of the survival function was employed. The log-rank test was used to assess the significance of differences between two or more survival curves. Univariate and multivariate Cox proportional hazard models were used to examine the prognostic value of various factors on patient survival. The analyses were performed in R 4.3.0 with the survival and survminer libraries, and in MATLAB R2007 with the statistical toolbox. All tests were performed at a 5% significance level.

## Results

### *ATM* and *TP53* aberrations tend to be exclusive

Genetic aberrations (i.e., deletions and/or mutations) of the *ATM* and *TP53* genes were examined in 187 consecutive adult patients with newly diagnosed MCL using FISH and NGS. The distribution of the detected aberrations of the two analyzed genes is shown in Table 1.

**Table 1:** Distribution of the types of aberrations of the *ATM* and *TP53* genes in the patient population. Abbreviations: *ATM*wt – *ATM* wild-type; del11 – deletion of 11q; mut*ATM* – mutation of *ATM*; *TP53*wt – *TP53* wild-type; del17 – deletion of 17p; mut*TP53* – mutation of *TP53*

		*TP53* status			
		***TP53*** **wt**	**del17**	**mut** ***TP53***	**del17 and mut** ***TP53***
*ATM* status	***ATM*** **wt**	54	8	13	31
	**del11**	15	3	4	8
	**mut** ***ATM***	12	1	3	3
	**del11 and mut** ***ATM***	31	1	0	0

Twenty-three (12%) patients had combined aberrations of both the *ATM* and *TP53* genes. The number of patients with deletions or mutations in both genes is significantly lower than expected if the aberrations were independent (*p* = 0.004). While mutations in the *TP53* gene were predominantly located in the DNA-binding domain, mutations in the *ATM* gene (mut*ATM*) were distributed throughout the gene, with no occurrence of the same mutation within the cohort.

**Fig. 1:**
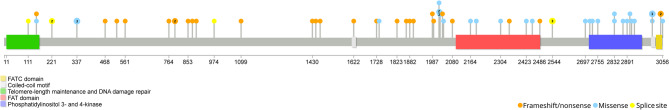
Lollipop plot of *ATM* gene variant distribution. The numbers represent the number of variants

Three (9%) out of 32 examined patients with available non-malignant DNA carried the rare germline mut*ATM* (g.108335845G > C, g.108345819G > A, and g.108251026_108251027delAG); all three were predicted to be pathogenic according to the prediction models. One patient had two additional rare germline *ATM* sequence variants (g.108251026_108251027delAG and g.108289652_108289655delAAAT); both were predicted to be pathogenic according to the prediction models. One patient with a rare germline *ATM* variant (g.108251026_108251027delAG) had an additional somatic variant (g.108289652_108289655delAAAT), which was also predicted to be pathogenic according to the prediction models. The variant distribution of the *ATM* gene is shown in Fig. 1.

Table2 shows the baseline characteristics of the entire population and compares patients with any *TP53* aberration to those with any *ATM* aberration. Interestingly, there are no significant differences in any of the analyzed markers.

**Table 2:** Characteristics of the entire population, *ATM* wild-type patients with any *TP53* aberration, and *TP53* wild-type patients with any *ATM* aberration. The table shows numbers of patients (percentages in brackets). The p-values result from comparing the *ATM* aberrated and *TP53* aberrated populations. Chi-square tests are used for binary markers, and Kruskal–Wallis tests are used for continuous markers. Abbreviations: PS ECOG *–* Eastern Cooperative Oncology Group performance status; MIPI – Mantle Cell Lymphoma International Prognostic Index; HDAC – high-dose cytarabine; ASCT – autologous stem cell transplant; CHOP – cyclophosphamide, doxorubicin, vincristine, prednisone; CR – complete remission; PR – partial remission; SD – stable disease; PD – progressive disease

	all patients (*n*=187)	*TP53* aberrated (*n*=52)	*ATM* aberrated (*n*=58)	*p*-value
Male	126 (67.4)	39 (75.0)	41 (70.7)	0.16
Median age, (range)	66.9 (30–87)	67.6 (30–87)	67.0 (42–82)	0.76
PS ECOG (0/1/2/3)	68 (36.4) / 87 (46.5) / 24 (12.8) / 8 (4.3)	22 (42.3) / 19 (36.5) / 6 (11.5) / 5 (9.6)	17 (29.3) / 30 (51.7) / 10 (17.2) / 1 (1.7)	0.33 (0–1 vs 2–3)
B-symptoms	78 (41.8)	22 (42.3)	28 (48.3)	0.87
Ki67 (≥30% / N/A)	47 (43.5) / 79 (42.2)	12 (63.2) / 33 (63.5)	14 (31.1) / 13 (22.4)	0.67
Morphology (class. / blast./pleom. / N/A)	50 (67.6) / 24 (32.4) / 113 (60.4)	12 (66.7) / 6 (33.3) / 34 (65.4)	18 (75) / 6 (25) / 34 (58.6)	0.71 (class. vs other)
MIPI (low / inter. / high)	27 (14.4) / 51 (27.3) / 109 (58.3)	5 (9.6) / 14 (26.9) / 33 (63.5)	9 (15.5) / 13 (22.4) / 36 (62.1)	0.86 (high vs other)
Induction (HDAC+ASCT / CHOP / Non-Anthracycline / obs.)	67 (35.8) / 92 (49.2) / 19 (10.2) / 9 (4.5)	13 (25.0) / 28 (53.8) / 9 (17.3) / 2 (3.8)	20 (34.5) / 35 (60.3) / 3 (5.2) / 0	0.87 (HDAC vs other)
Maintenance rituximab	104 (55.6)	22 (42.3)	42 (72.4)	0.79
Response (CR / PR / SD+PD)	106 (56.7) / 38 (20.3) / 26 (13.9)	25 (48.1) / 12 (23.1) / 7 (13.4)	37 (63.8) / 12 (20.7) / 6 (10.3)	0.39 (CR+PR vs other)

### *ATM* deletion correlates with shorter PFS in the subcohort of patients with wild-type *TP53*, but not in the entire cohort

**Fig. 2:**
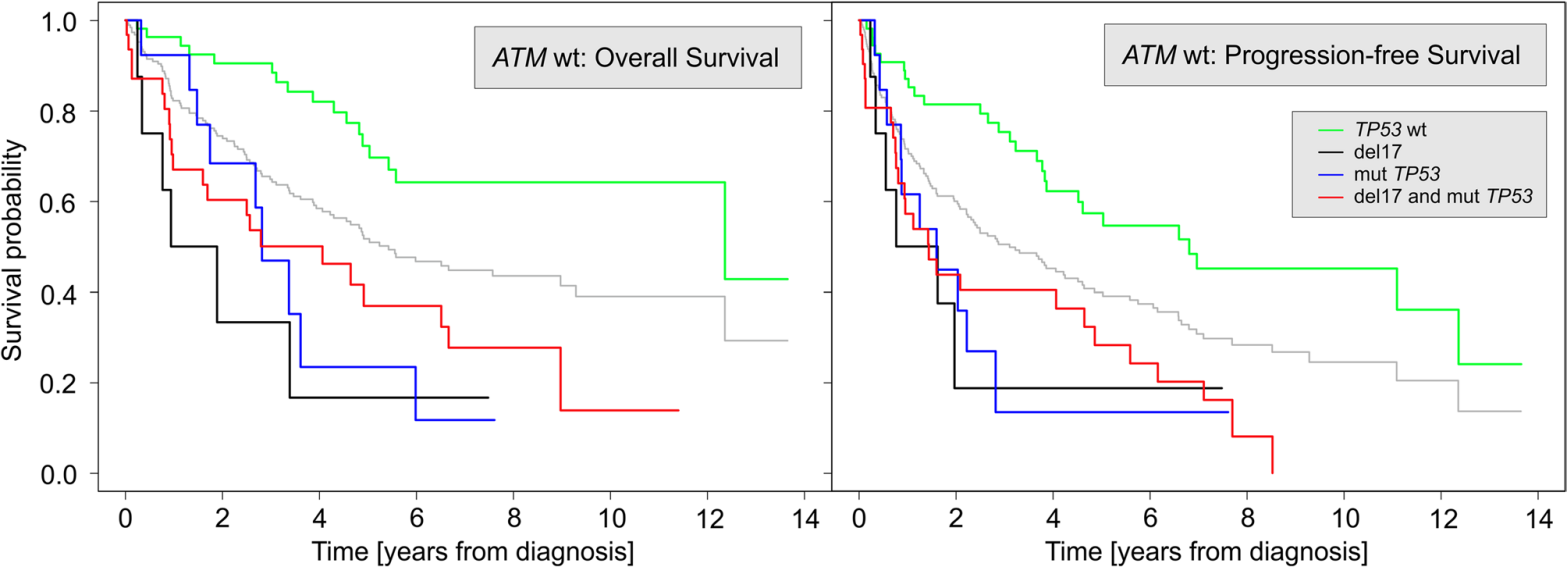
OS (left) and PFS (right) of the 106 patients with an unaffected *ATM* gene (*ATM*wt). The *TP53* aberration status is color-coded. The gray lines in the background correspond to the OS of the entire cohort (left) and PFS of the entire cohort (right) for easier comparison. The difference in survival probabilities among the four groups is significant at *p* < 0.001 for both OS and PFS

In the entire cohort, *TP53* aberrations (either mutations or deletions) were associated with worse outcomes, namely shorter survival (*p* < 0.005 for both OS and PFS). Similarly, in patients with an unaffected *ATM* gene (*ATM*wt), *TP53* aberrations were significantly correlated with shorter PFS and OS compared to *TP53* wild-type (*TP53*wt) patients. Of note, no survival difference was observed between patients with del17 and mut*TP53*. Regarding OS (left panel of Fig. 2), the *TP53*wt cohort had the longest survival (median survival 12.4 years from diagnosis). Del17 seems to be associated with the worst outcome (median survival 17.0 months). Mut*TP53* exhibited a median survival of 2.8 years months, and mut*TP53* together with del17 exhibited a median survival of 4.1 years. The difference in the survival probabilities among the four groups is significant at *p* < 0.001. The situation is similar for PFS (right panel of Fig. 2), with an even clearer pattern. The difference between the *TP53*wt group and the remaining three groups is even more distinct. The median survival times are 6.8 years for *TP53*wt, 1.2 years for del17, 1.6 years for mut*TP53*, and 1.4 years for del17 + mut*TP53*. The difference is significant at *p* < 0.001; the wild-type group differs significantly from the other three groups. For more detailed results on the impact of *TP53* aberrations on survival, see the Supplementary Materials.

**Fig. 3:**
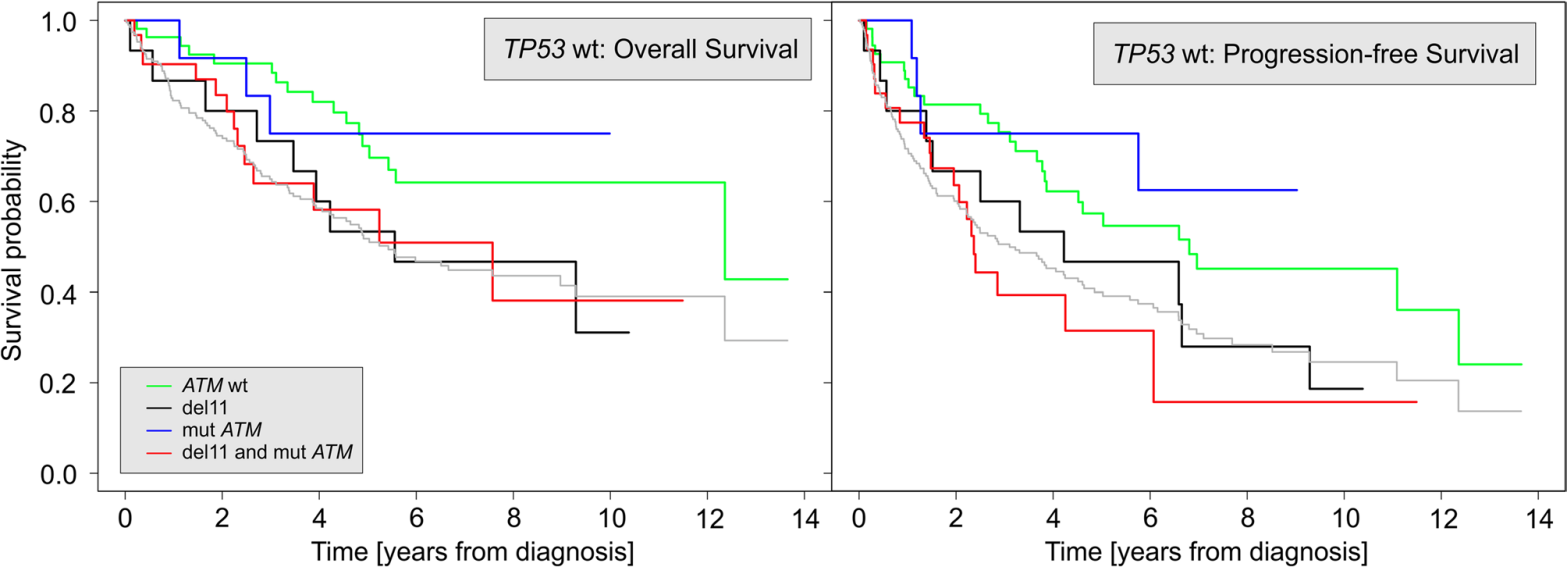
OS (left) and PFS (right) of the 112 patients with an unaffected *TP53* gene. The *ATM* gene aberration status is color-coded. The gray lines in the background correspond to OS of the entire cohort (left) and PFS of the entire cohort (right) for easier comparison. Surprisingly, the *ATM* wild-type group is *not* the one with the best outcome. Abbreviations: *ATM*wt – *ATM* wild-type; del11 – deletion of 11q; mut*ATM* – mutation of *ATM*; *TP53*wt – *TP53* wild-type; del17 – deletion of 17p; mut*TP53* – mutation of *TP53*

In contrast, *ATM* aberrations were not prognostic when analyzed for the entire cohort (*p* = 0.19 for OS and *p* = 0.36 for PFS) (see Supplementary Material). However, since we and others have demonstrated that *ATM* and *TP53* aberrations tend to be exclusive in MCL, we analyzed the prognostic significance of *ATM* aberrations also in the subcohort of patients with *TP53*wt (Fig. 3).

In the subcohort of patients with *TP53*wt, PFS did not decrease below 50% for patients with mut*ATM* during the study period. However, PFS reached 50% for *ATM*wt, deletion of 11q (del11), and the del11 + mut*ATM* combination after 6.8, 4.2, and 2.4 years from diagnosis, respectively.

Using the *ATM*wt group as a reference and computing the hazard ratios (HRs) for the remaining three groups, we found that only the del11 + mut*ATM* group had a significantly worse survival (HR = 2.25, *p* = 0.01). The other two groups did not differ statistically from the reference group. Importantly, del11 + mut*ATM* remained significant in a multivariate Cox analysis (HR = 2.15, *p* = 0.03), as did age. This suggests that, within the *TP53*wt population, the type of *ATM* aberration has almost no significant impact on PFS. After controlling for covariates, the most affected group (del11 + mut*ATM*) had slightly worse PFS.

In the subcohort of patients with *TP53*wt, OS did not decrease below 50% for patients with mut*ATM* during the study period. However, OS reached 50% for *ATM*wt, del11, and del11 + mut*ATM* after 12.4, 5.6, and 7.6 years from diagnosis, respectively.

When using the *ATM*wt *group* as a reference and computing HRs for the remaining three groups, only the del11 + mut*ATM* group was found to have significantly worse survival (HR = 2.14, *p* = 0.04). The other two groups did not differ statistically from the reference group. Unlike PFS, age was the only significant predictor of OS in a Cox multivariate analysis.

### Prognostic significance of ATM mutation burden and percentage of del11

The median mut*ATM* burden (VAF) was 25.5% (range, 5.0%–89.7%), and the median del11 cancer clonal fraction was 31.0% (range, 8.0%–93.0%). These two variables were strongly correlated in patients with both aberrations (c = 0.53, *p* = 0.01). Of note, a high mut*TP53* burden was associated with a low mut*ATM* burden (c = −0.29, *p*= 0.06). This is likely an example of Berkson’s paradox (Berkson 1946).

**Fig. 4:**
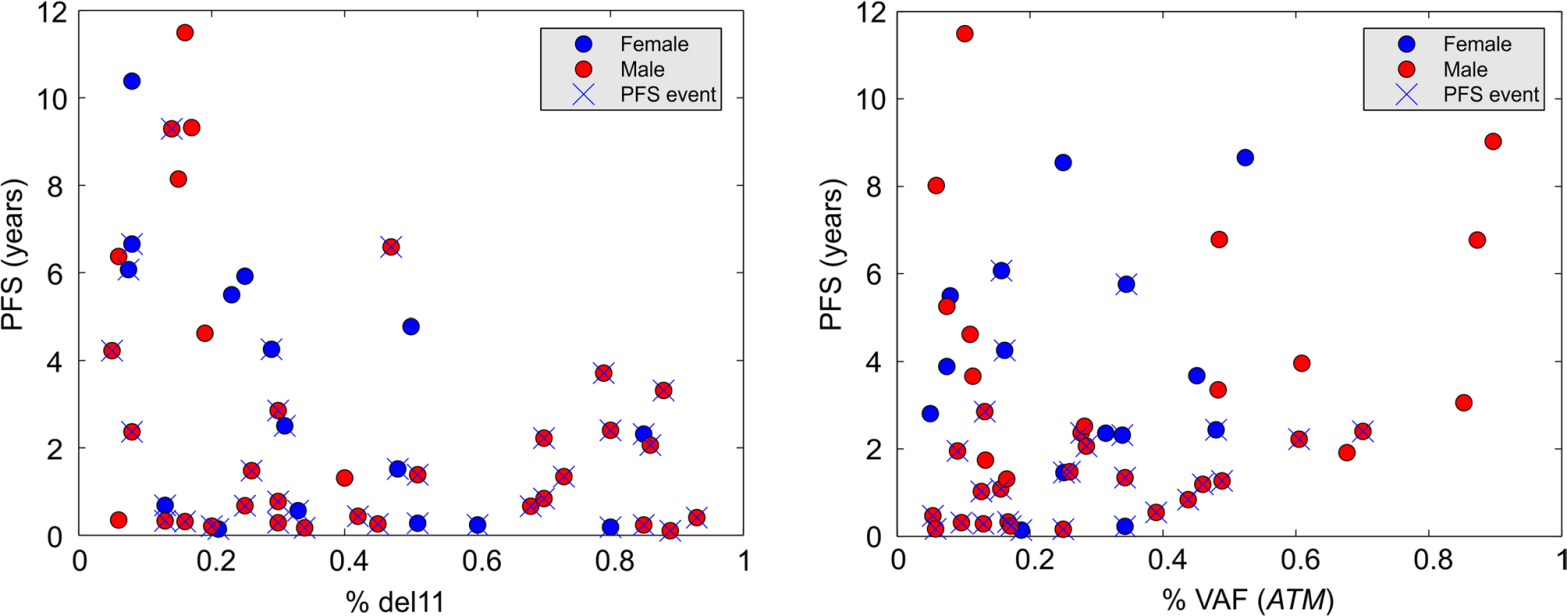
The associations between the percentage of del11 and progression-free survival (left panel), and between the *ATM* gene mutation burden and progression-free survival (right panel). Each dot represents a single patient. Gender is color-coded. Patients with a PFS event are marked by a cross

A high percentage of del11 correlated with shorter PFS (c = −0.37, *p* = 0.008) (Fig. 4A). Almost all patients with a del11% greater than 30% experienced an event, and their PFS was shorter than four years. Conversely, nearly all patients with PFS exceeding four years had a del11% below 30%. This effect was not confounded by age since the correlation between age and del11% was modest (c = 0.33, *p* = 0.02).

In contrast, a high mut*ATM* burden (i.e., a high VAF of mut*ATM*) was associated with a trend toward longer PFS (Fig. 4B).

Although the correlation is not statistically significant (*p* = 0.38), an interesting pattern could be deduced from Fig. 4B. All patients with mut*ATM* VAF greater than 50% had PFS exceeding two years, and almost none of them experienced an event. Conversely, many patients with mut*ATM* VAF below 50% experienced a PFS event within two years from diagnosis. This further supports the above observation that mut*ATM* seems to be a protective factor, as patients with mut*ATM* exhibit longer PFS than those with *ATM*wt.

### Analysis of aberrant regions of 11q

Array CGH analysis was available for 42 patients in our cohort. Eighteen patients (43%) showed at least one 11q aberration. Overall, we identified 29 regions of 11q deletion, seven regions of 11q gain, and one copy-neutral loss of heterozygosity. In 12 patients, the *ATM* gene was included in the deleted regions, which varied in range. The smallest commonly deleted region (SCDR), which was 8 Mbp, was localized at 11q22.3-q23.2 (106.2–114.2 Mbp). In addition to *ATM*, it contained another 56 annotated genes (www.omim.org).

## Discussion

MCL is characterized by genome instability and a predisposition to large-scale cytogenetic changes. The present study focused on the prognostic role of *ATM* gene aberrations in MCL in the entire cohort and in patients with *TP53*wt. The prevalence rates of *ATM* and *TP53*aberrations in our cohort were consistent with previously published data (Malarikova et al. 2020; Camacho et al. 2002; Mareckova et al. 2019; Sakhdari et al. 2019; Eskelund et al. 2017; Obr 2020; Obr et al. 2018; Koff et al. 2022; Meissner et al. 2013; Zhang et al. 2014; Rossi et al. 2015; Halldórsdóttir et al. 2011). We confirmed that simultaneous*TP53* and *ATM*mutations and deletions are very rare, which also corresponds with previously published data (Malarikova et al. 2020; Mareckova et al. 2019; Koff et al. 2022).

Aberrations in the *TP53* gene play a crucial role in lymphomagenesis, leading to chemoresistance and shorter survival, irrespective of the therapeutic regimen used. In our study, mut*TP53* and del17 in *ATM*wt patients were both significantly associated with shorter PFS and OS. Despite the long inclusion period of our study (2006–2022) and the historically heterogeneous therapeutic approaches, we did not observe any improvement in prognosis attributable to treatment advances. Our results thus confirmed previously published data (Malarikova et al. 2020; Eskelund et al. 2017; Obr 2020; Obr et al. 2018; Koff et al. 2022; Delfau-Larue et al. 2015; Aukema et al. 2018). Unlike other reports (Eskelund et al. 2017; Obr et al. 2018; Halldórsdóttir et al. 2011), we did not observe a differential prognostic impact of del17 and mut*TP53.* This could be explained by the relatively small number of *ATM*wt patients with *TP53* aberrations and the presence of more biallelic del17, with consequent complete inactivation of the *TP53*, in our cohort. We can also hypothesize that the high rate of overlap between *TP53* and *ATM* aberrations in our cohort (23 patients) causes higher genome instability and deletes relatively better outcomes for MCL patients with del17 compared to those with mut*TP53*.

In patients with available buccal swab samples, the ratio of germline mutations in the *ATM*genes in our study (9%) was lower than in a study by Mareckova et al. (18%) or in chronic lymphocytic leukemia (CLL) (Mareckova et al. 2019; Lampson et al. 2023). All germline mut*ATM*in our patients were pathogenic, consistent with recently published data (Petrackova et al. 2022). Although the prevalence of germline mut*ATM*in our analysis was lower than in Mareckova´s cohort, 9% still exceeded the 1% rate in the general population (Swift et al. 1991). According to other publications, germline*ATM* mutations are more frequent in MCL patients than in healthy people. It appears that germline mut*ATM*increases the risk of lymphoproliferative disorders, such as CLL, non-Hodgkin lymphoma (Lampson et al. 2023; Mavrou et al. 2008; Suarez et al. 2015), and especially MCL (Usui et al. 2022). The observed prevalence of germline mut*ATM* in MCL warrants the consideration of routine germline testing, particularly in patients with a family history of cancer. In addition, it would be beneficial to strengthen surveillance of patients carrying germline mut*ATM*, as a recent CLL study in showed a higher incidence of secondary cancers in these patients (Petrackova et al. 2025).

Because we and others have demonstrated that *ATM* and *TP53* are rarely found together in MCL, the prognostic role of *ATM* gene aberrations was analyzed both in the entire cohort and in a subcohort of patients with *TP53*wt.

Interestingly, we observed slightly longer PFS as well as OS, albeit not significantly, in patients with mut*ATM* compared to those with *ATM*wt. These results may be influenced by the small number of the patients, but they suggest a protective role of mut*ATM*. Similar results were relatively recently published by Chinese investigators. The authors observed enriched antitumor pathways and more abundant tumor-infiltrating cytotoxic lymphocytes in endometrial tumors with mut*ATM*. The analyzed cohort of MCL patients with the mut*ATM* experienced longer survival compared to those with wild-type *ATM*(Sun et al. 2020). The higher abundance of cytotoxic T lymphocytes with an antitumor effect in mut*ATM* MCL could explain the longer survival in our analyzed cohort as well. On the other hand, patients in our study with a simultaneous *ATM* mutation and deletion had poorer PFS and OS. The occurrence of mutation or deletion of *ATM* alone was not associated with significantly shorter survival. The lack of an effect of mut*ATM*on shorter survival in our study is consistent with previously published data (Camacho et al. 2002; Mareckova et al. 2019; Greiner et al. 2006; Eskelund et al. 2017). However, a recent paper reported that mut*ATM*was associated with shorter PFS and OS (Koff et al. 2022). Authors of the aforementioned study analyzed solely the*ATM* mutation profile without FISH examination of the samples. We cannot rule out that the presence of del11 in the aforementioned study population with mut*ATM* could have impacted the survival outcomes negatively.

A high mut*TP53* burden was correlated with a low burden of mut*ATM*. This finding underlines the exclusivity of mut*TP53* and mut*ATM* discussed above. Namely, different second hits in MCL may shape different mutational profiles in patients with *TP53* and *ATM* aberrations.

The present study strongly suggests that del11 and mut*ATM* have dramatically different impacts on the survival prognosis of MCL patients with *TP53*wt. First, del11, but not mut*ATM*, was associated with shorter PFS. Second, a high frequency of del11 correlated with shorter PFS. Third, patients with a high burden of mut*ATM* had a trend toward longer PFS. Based on these findings, we conclude that a high frequency of del11 is an independent predictor of early relapse, progression, or death. Conversely, a high mut*ATM* burden appears to play a protective role in terms of PFS, at least in the context of the currently used immunochemotherapy.

The adverse prognostic impact of different types of *ATM* aberrations has been previously discussed in the context of patients with CLL. It was reported that a single mut*ATM*does not play a crucial role in chemoresistance or survival (Jiang et al. 2016). In contrast, the combined presence of mut*ATM*and del11 resulted in shorter survival and an insufficient response to alkylating agents and purine analogs (Skowronska et al. 2012).

Concerning molecular substantiation, we propose that monoallelic inactivation of *ATM* due to gene mutation may not be as important as complete loss of gene function due to biallelic del11 or a combination of a monoallelic del11 and mut*ATM* on the second allele. Similar findings were observed in CLL patients with biallelic involvement of *ATM*who had an inadequate response to treatment and shorter OS (Lozano-Santos et al. 2017).

Unlike mut*ATM*, del11 results in genetic inactivation of many genes besides *ATM*. Specifically, the long arm of chromosome 11 contains 2,057 genes (47undefined ). In the present study, we used array CGH to define the SCDR on 11q. In addition to the*ATM* gene, this deleted region contained genes involved in various human diseases and cancers, for example, *ALKBH8*, *ACAT1*, *CUL5*, *NPAT*, *EXPH5*, *RDX*,* BTG4*, *PPP2R1B*, *ALG9*, *CRYAB*, *DLAT*, *SDHD*, *PTS*, *TTC12*, and *ZBTB16*. However, none of the genes were reported as candidate genes associated with MCL. *NPAT* (nuclear protein, ataxia-telangiectasia locus), a gene located in close proximity to *ATM*, regulates the cell cycle and histone gene transcription. It encodes a strongly conserved 1,427 amino acid protein containing nuclear localization signals and target sites for phosphorylation by cyclin-dependent protein kinases associated with the transcription factor E2F. *NPAT* is expressed in all human tissues and appears essential for cell maintenance. The *ATM* and *NPAT*genes share a promoter region and influence the expression of each other (Imai et al. 1996). Given that all cases with*ATM* deletion, as detected by FISH, typically carry co-deletion of *ATM* with *NPAT*, leading to haploinsufficiency, it is theoretically possible that *NPAT* reduces the resistance of cells to DNA stress. Other genes implicated in deregulation of the cell cycle and apoptosis regulators located within the SCDR are, for example, *CUL5* (ubiquitin-dependent apoptosis regulation) and *PPP2R1B* (component of the cell cycle- and apoptosis-regulating PP2A). Their role in the pathogenesis of CLL was studied by German authors who observed significant down-regulation of *NPAT* and *CUL5* in CLL cases with del11. They also observed reduced *PPP2R1B* transcript levels in a subset of CLL cases. Alternative splicing of *PPP2R1B*correlated with reduced activity of protein phosphatase 2 A (Kalla et al. 2007). Decreased expression of*CUL5* might contribute to disease progression in CLL patients, as *CUL5* is involved in regulating *TP53* stability and ubiquitin-dependent control of apoptosis. Interestingly, the *BIRC3* gene, that plays a pivotal role in regulating NF-κB signaling and apoptosis, was not included in the SCDR. Aberrations in *BIRC3*are associated with worse outcomes in CLL patients and resistance to ibrutinib, a covalent inhibitor of Bruton’s tyrosine kinase (BTK) (Jiang et al. 2016). A deep sequencing investigation of chromosome 11 and its lost region is needed to better understand the prognostic value of del11 range in MCL.

Based on the available results, we can assume that patients with *TP53* aberrations have an inadequate response to DNA damage and that *ATM* inactivation by deletion is redundant. In *TP53*wt patients, monoallelic or biallelic deletion of *ATM* may silently exacerbate genetic instability. The loss of *ATM* down-regulates otherwise functional p53, impairing apoptosis and G1/S checkpoint response. Furthermore, the *ATM* gene has been shown to activate as soon as cell damage occurs, triggering cell repair and *TP53*activity (Sturm et al. 2003). Del11 is likely sufficient to drive pathogenesis and poor prognosis, particularly in the context of an intact*TP53* pathway that subsequently fails to be properly activated.

Understanding the role of *ATM* in the DNA damage response suggests that patients with del11 may benefit from therapies targeting defective DNA repair pathways. Poly(ADP-ribose) polymerase (PARP) inhibitors, which exploit synthetic lethality in *ATM*-deficient cells, have demonstrated efficacy in treating solid tumors with *ATM*aberrations, such as prostate and breast cancer (Fong et al. 2009). The PARP inhibitors olaparib and niraparib are currently being investigated for use in CLL, MCL, and other relapsed or refractory hematologic malignancies (ClinicalTrials.gov studies NCT03317392 and NCT04267913). Similarly, ataxia telangiectasia and Rad3-related protein (ATR) inhibitors (e.g., ceralasertib/AZD6738) have shown promising activity in preclinical models by selectively targeting*ATM*-deficient cells (Yazinski et al. 2017). Their effects on hematologic cancers are currently being evaluated in clinical trials (ClinicalTrials.gov studies NCT02264678 and NCT04497116). The increased genomic instability caused by*ATM* dysfunction may also render these patients more susceptible to novel immunotherapies, including immune checkpoint inhibitors. However, further clinical validation is needed. BCL2 and, in particular, BTK inhibitors have revolutionized the treatment of MCL, despite not being directly linked to *ATM* biology. Based on limited data on their efficacy in *ATM*-deficient patients, a combination of venetoclax and rituximab may be effective for this unfavorable patient subgroup (Seymour et al. 2022). Chimeric antigen receptor (CAR) T-cell therapy has shown high efficacy in various malignancies, including MCL (Huang et al. 2023). However, data on its effectiveness in*ATM* aberrant diseases are lacking. Given the higher genomic instability caused by *ATM* dysfunction, an indirect impact on the immune system or tumor microenvironment potentially influencing the efficacy of CAR T-cell therapy can be anticipated. Furthermore, impaired *ATM*function may lead to the accumulation of chromosomal lesions and increased leukemogenic potential (Rozenbaum et al. 2024). To the best of our knowledge, none of the current clinical trials specifically select or stratify patients based on*ATM* status. However, this topic is complex and warrants further investigation.

Beyond the currently approved targeted therapies, patients harboring del11 and mut*ATM*, or those with a high del11 burden, may benefit from novel agents that exploit vulnerabilities in DNA repair mechanisms, such as PARP and ATR inhibitors. Although these therapies are still under clinical investigation, they are supported by a mechanistic rationale rooted in synthetic lethality. In addition, CAR T-cell therapy holds promise for this high-risk subgroup, but it remains insufficiently explored. Future clinical trials should consider stratifying patients by *ATM* status to clarify its predictive value for treatment response.

## Conclusions

In conclusion, considering the mutually exclusive nature of mutations and deletions in *TP53* and *ATM*, we strongly recommend that patients exhibiting del11 and *ATM* mutations, or those with a high prevalence of del11, be considered for innovative therapeutic approaches, regardless of their *TP53* status.

## Supplementary Information

Supplementary Material 1.

## Data Availability

All study data are available from the main author of the manuscript upon reasonable request.

## Abbreviations

MCL: mantle cell lymphoma
ATM: ataxia-telangiectasia mutated
AT: ataxia-telangiectasia
TP53: tumor protein p53
del17: deletion of 17p
mutTP53: mutated TP53
BM: bone marrow
PB: peripheral blood
FISH: fluorescence in situ hybridization
NGS: next-generation sequencing
CGH: comparative genomic hybridization
VAF: variant allele frequency
OS: overall survival
PFS: progression-free survival
mutATM: mutated ATM
ATMwt: ATM wild-type
TP53wt: TP53 wild-type
del11: deletion of 11q
HR: hazard ratio
SCDR: smallest commonly deleted region
CLL: chronic lymphocytic leukemia
NPAT: nuclear protein, ataxia-telangiectasia locus
BTK: Bruton’s tyrosine kinase
PARP: poly(ADP-ribose) polymerase
ATR: ataxia telangiectasia and Rad3-related protein
CAR: chimeric antigen receptor
