# Supplementary material for: Fundamental prognostic difference of ATM gene mutation and deletion in newly diagnosed mantle cell lymphoma
Source: Mol Med. 2025 Sep 29;31:306. doi: 10.1186/s10020-025-01376-2 (PMC12481964; doi:10.1186/s10020-025-01376-2)
Supplement: Supplementary file 1 — Supplementary Material 1. [file 10020_2025_1376_MOESM1_ESM.docx]

**Fundamental prognostic difference of ATM gene mutation and deletion in newly diagnosed mantle cell lymphoma**

*Patients with the wild-type ATM gene: overall survival*

Let us describe the effect of *TP53* aberration (in the context of all the other variables listed in Table 2) on OS and PFS in the population with a normal (wild-type) *ATM* gene. Figure 2 shows the Kaplan–Meier estimates of the survival functions according to *TP53* aberration status.

Using the *TP53* wild-type (wt*TP53*) group as a reference, and computing the hazard ratios (HRs) for the remaining three groups with *TP53* aberrations, we found that all the HRs were significantly (p < 0.001) greater than one. Specifically, the HR was 5.87 for del17, 4.11 for mut*TP53*, and 3.45 for del17 + mut*TP53*. However, the confidence intervals overlap, indicating no significant difference in OS among the three groups with *TP53* aberration.

Let us further examine the strength of the *TP53* aberration status as a predictor of OS. We built a multivariate Cox PH model by adding age at diagnosis, gender, presence of B-symptoms, and MIPI (categorical) to the model. In such a model, age and B-symptoms have a significant effect on OS; however, the significant dependence of OS on the *TP53* aberration status is retained. Specifically, the HR was 6.4 for del17, 3.71 for mut*TP53*, and 2.58 for del17 + mut*TP53* (p < 0.01 in all three cases). Thus, the aberration status of *TP53* remains a significant predictor of OS even after adjusting for covariates.

*Patients with the wild-type ATM gene: progression-free survival*

Using the wt*TP53* group as a reference, and computing the HRs for the remaining three groups with *TP53* aberrations, we found that all the HRs were significantly (p < 0.01) greater than one. Specifically, the HR was 3.42 for del17, 3.30 for mut*TP53*, and 2.84 for del17 + mut*TP53*. However, the confidence intervals overlap, indicating no significant difference in PFS among the three groups with *TP53* aberration.

Then we built a multivariate Cox PH model by adding age at diagnosis, gender, presence of B-symptoms, and MIPI (categorical) to the model. In such a model, age and B-symptoms have a significant effect on PFS; however, the significant dependence of PFS on the *TP53* aberration status is retained. Specifically, the HR was 3.57 for del17, 4.78 for mut*TP53*, and 2.14 for del17 + mut*TP53* (p < 0.05 in all three cases). Thus, the aberration status of *TP53* remains a significant predictor of PFS even after adjusting for covariates.

*ATM aberrations in the entire cohort: overall survival and progression-free survival*

*ATM* aberrations were not prognostic when analyzed for the entire cohort, as shown in Supplemental Figure 1.


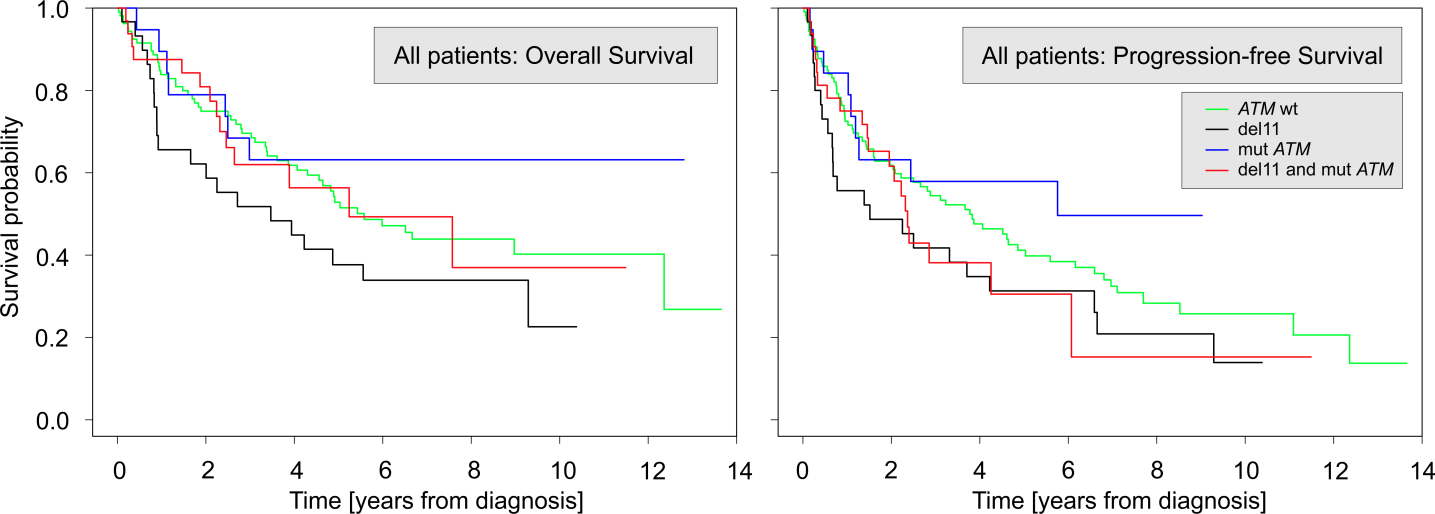


Suppl. Figure 1. OS (left) and PFS (right) for the entire cohort. The aberration status of *ATM* is color-coded.
